# The association between renal recovery after acute kidney injury and long-term mortality after transcatheter aortic valve replacement

**DOI:** 10.1371/journal.pone.0183350

**Published:** 2017-08-17

**Authors:** Charat Thongprayoon, Wisit Cheungpasitporn, Narat Srivali, Wonngarm Kittanamongkolchai, Ankit Sakhuja, Kevin L. Greason, Kianoush B. Kashani

**Affiliations:** 1 Division of Nephrology and Hypertension, Mayo Clinic, Rochester, Minnesota, United States of America; 2 Division of Pulmonary and Critical Care Medicine, Mayo Clinic, Rochester, Minnesota, United States of America; 3 Department of Cardiovascular Surgery, Mayo Clinic, Rochester, Minnesota, United States of America; University of Sao Paulo Medical School, BRAZIL

## Abstract

**Background:**

This study aimed to examine the association between renal recovery status at hospital discharge after acute kidney injury (AKI) and long-term mortality following transcatheter aortic valve replacement (TAVR).

**Methods:**

We screened all adult patients who survived to hospital discharge after TAVR for aortic stenosis at a quaternary referral medical center from January 1, 2008, through June 30, 2014. An AKI was defined as an increase in serum creatinine level of 0.3 mg/dL or a relative increase of 50% from baseline. Renal outcome at the time of discharge was evaluated by comparing the discharge serum creatinine level to the baseline level. Complete renal recovery was defined as no AKI at discharge, whereas partial renal recovery was defined as AKI without a need for renal replacement therapy at discharge. No renal recovery was defined as a need for renal replacement therapy at discharge.

**Results:**

The study included 374 patients. Ninty-eight (26%) patients developed AKI during hospitalization: 55 (56%) had complete recovery; 39 (40%), partial recovery; and 4 (4%), no recovery. AKI development was significantly associated with increased risk of 2-year mortality (hazard ratio [HR], 2.20 [95% CI, 1.37–3.49]). For patients with AKI, the 2-year mortality rate for complete recovery was 34%; for partial recovery, 43%; and for no recovery, 75%; compared with 20% for patients without AKI (*P* < .001). In adjusted analysis, complete recovery (HR, 1.87 [95% CI, 1.03–3.23]); partial recovery (HR, 2.65 [95% CI, 1.40–4.71]) and no recovery (HR, 10.95 [95% CI, 2.59–31.49]) after AKI vs no AKI were significantly associated with increased risk of 2-year mortality.

**Conclusion:**

The mortality rate increased for all patients with AKI undergoing TAVR. A reverse correlation existed for progressively higher risk of death and the extent of AKI recovery.

## Introduction

Transcatheter aortic valve replacement (TAVR) is a revolutionary, relatively new catheter-based technique for treating patients with severe aortic stenosis who are at high risk for a surgical aortic valve replacement [1]. In addition, recent studies have also suggested the potential use of TAVR for patients at intermediate [2,3] and low risk [4], as well as for a subset of patients whose aortic stenosis was more technically challenging to repair surgically [5,6]. Although TAVR is considered less invasive than surgical repair, patients undergoing TAVR usually have more comorbidities [7]. Nearly 25% of patients die within the first year after the procedure, despite the recent advances and success of TAVR technology [1,8–10].

Acute kidney injury (AKI) after TAVR is common (reported for up to 57% of patients) [7,11,12] and is independently associated with a higher risk of mortality [12,13]. Recently, the impact of renal recovery after AKI on patient outcomes has been comprehensively described [14–16]. Recovery status has been reported to affect long-term outcomes of critically ill patients [15], and patients who have complete renal recovery have significantly better survival rates than patients who have only partial renal recovery or no recovery [15,17,18]. Poor baseline renal function and AKI have been shown to predict outcomes after TAVR [11,19]; however, little is known about the impact of renal recovery following AKI. Therefore, we aimed to examine the association between renal recovery status at hospital discharge after AKI and long-term mortality following TAVR.

## Materials and methods

### Patient population

A retrospective cohort study was conducted at Mayo Clinic Hospital in Rochester, Minnesota, from January 1, 2008, through June 30, 2014. Adult patients (≥18 years) were included who survived to hospital discharge after having a TAVR. Patients were excluded if they had dialysis within 14 days before the procedure or they did not provide research authorization. This study was approved by the Mayo Clinic Institutional Review Board and informed consent was waived for patients who provided research authorization.

### Data collection

Manual and automated retrieval of institutional electronic health records were performed to collect demographic, clinical, laboratory, echocardiographic, procedural, and postprocedural data. The estimated percentage for 30-day mortality was determined by using the Society of Thoracic Surgeons risk score, which calculates risk via a model that uses patient demographic characteristics, preoperative clinical characteristics, and type of procedure being performed [20–22]. The Chronic Kidney Disease Epidemiology Collaboration equation was used to calculate the estimated glomerular filtration rate (eGFR) [23].

### Definition of AKI and renal recovery

AKI was defined according to the criteria of the Valve Academic Research Consortium-2 (VARC-2), ie, an increase in serum creatinine of at least 0.3 mg/dL within 48 hours or a relative increase of at least 50% within 7 days from baseline [24]. The most recent outpatient value from 7 to 180 days before the procedure was used as baseline. If that value was not available, the lowest serum creatinine value within 5 days before the procedure was used.

Renal outcome was assessed at the time of hospital discharge by comparing the serum creatinine at discharge to the serum creatinine at baseline. Complete renal recovery was defined as no AKI at patient discharge, whereas partial renal recovery was defined as AKI without the need for renal replacement therapy at discharge. No renal recovery was defined as a need for renal replacement therapy at discharge.

### Clinical outcome

The primary outcome was all-cause mortality 2 years after hospital discharge. We reviewed the Mayo Clinic electronic health records and the Social Security Death Index to determine patient deaths [25]. The secondary outcomes included the change in eGFR at 2 years after discharge and initiation of dialysis.

### Statistical analysis

Continuous variables were described as mean±standard deviation and were compared using analysis of variance. Categorical variables were reported as counts with percentages and were compared using the χ^2^ test. Kaplan-Meier analysis was used to generate curves for patient survival after hospital discharge, which were compared using the the log-rank test. Cox proportional hazards regression analysis was used to assess the association between degree of renal recovery and 2-year mortality after hospital discharge, adjusting for a priori defined covariates, Society of Thoracic Surgeons risk score, age, eGFR, and arterial approach. Results of 2-sided tests with a *P* value less than .05 were considered statistically significant. All analyses were performed with JMP statistical software (SAS Institute Inc, Cary, NC).

## Results

We screened 390 patients who underwent TAVR for aortic stenosis during the study period. Sixteen patients were excluded: 11 died in the hospital, 3 received dialysis before their procedure, and 2 did not provide research authorization. Therefore, 374 patients were included in the study.

The baseline characteristics of the study cohort are shown in Table 1. Their mean age was 81±8 years, and 56% were men. The mean Society of Thoracic Surgeons risk score was 8.5±6.2. The mean eGFR was 55±21 mL/min/1.73m^2^. The approach to TAVR was transfemoral in 50% of patients, transapical in 45%, and transaortic in 5%. Table 1 also includes the clinical characteristics of the patients according to the degree of renal recovery.

**Table 1:** Baseline characteristics^a^.

Characteristics	Complete Recovery (n = 55)	Partial Recovery (n = 39)	No Recovery (n = 4)	No AKI (n = 276)	*P* Value
STS risk score	10.1±6.3	8.6±5.2	11.2±3.7	8.1±6.3	.12
Age, y	82±8	81±7	82±4	81±8	.77
Male sex	27 (49)	26 (67)	3 (75)	152 (55)	.32
White	51 (93)	39 (100)	3 (75)	269 (97)	.01
BMI (kg/m^2^)	30.7±7.3	30.0±6.6	34.8±9.8	30.3±7.6	.65
eGFR (mL/min/1.73 m^2^)	47±20	50±26	32±14	58±19	< .001
NYHA class III-IV	51 (93)	34 (87)	4 (100)	234 (85)	.37
Comorbidity					
Diabetes mellitus	22 (40)	24 (62)	3 (75)	104 (38)	.02
Hypertension	51 (93)	37 (95)	4 (100)	246 (89)	.54
Dyslipidemia	48 (87)	38 (97)	4 (100)	245 (89)	.31
Myocardial infarction	15 (27)	18 (46)	3 (75)	98 (36)	.10
Congestive heart failure	36 (65)	21 (54)	4 (100)	152 (55)	.16
Stroke	15 (27)	14 (36)	1 (25)	79 (29)	.80
Peripheral vascular disease	35 (64)	23 (59)	4 (100)	157 (57)	.29
Anemia	2 (4)	1 (3)	1 (25)	6 (2)	.04
Chronic lung disease	33 (60)	25 (64)	2 (50)	174 (63)	.92
Smoking within 1 y	1 (2)	2 (5)	0 (0)	8 (3)	.80
Prior cardiac intervention					
Percutaneous coronary intervention	25 (45)	28 (72)	4 (100)	135 (49)	.01
Cardiac surgery	21 (38)	16 (41)	2 (50)	135 (49)	.45
CABG	21 (38)	15 (38)	2 (50)	122 (44)	.78
Valve surgery	14 (25)	11 (28)	1 (25)	56 (20)	.63
Aortic valve surgery	1 (2)	0 (0)	0 (0)	9 (3)	.64
Echocardiographic finding					
Ejection fraction	56±13	55±13	39±5	57±14	.05
Aortic valve gradient	46±16	48±12	40±9	49±14	.27
Aortic valve insufficiency	33 (60)	19 (49)	2 (50)	149 (54)	.74
Mitral valve dysfunction	45 (82)	27 (69)	3 (75)	216 (78)	.53
Preoperative medication					
ACE inhibitor/AR blocker	22 (40)	15 (38)	1 (25)	115 (42)	.90
β-Blocker	33 (60)	29 (74)	4 (100)	192 (70)	.22
Statin	38 (69)	29 (74)	3 (75)	205 (74)	.88
Aspirin	35 (64)	28 (72)	4 (100)	212 (77)	.13
Normal sinus rhythm	34 (62)	28 (72)	2 (50)	205 (74)	.21
Elective surgery	50 (91)	38 (97)	3 (75)	266 (96)	.06
Arterial approach					.003
Transfemoral	17 (31)	13 (33)	2 (50)	156 (57)	
Transapical	36 (65)	24 (62)	2 (50)	105 (38)	
Transaortic	2 (4)	2 (5)	0 (0)	15 (5)	
Surgery duration, min	132±68	125±48	113±42	126±47	.81
RBC transfusion	21 (38)	19 (49)	4 (100)	78 (28)	.001
Intra-aortic balloon pump	1 (2)	0 (0)	1 (25)	1 (0.3)	< .001

Abbreviations: ACE, angiotensin-converting enzyme; AR, angiotensin II receptor; BMI, body mass index; CABG, coronary artery bypass graft surgery; eGFR, estimated glomerular filtration rate; NYHA, New York Heart Association; RBC, red blood cell; STS, Society of Thoracic Surgeons.

^a^ Continuous variables are reported as mean ± SD, categorical variables as count (percentage).

**Fig 1:**
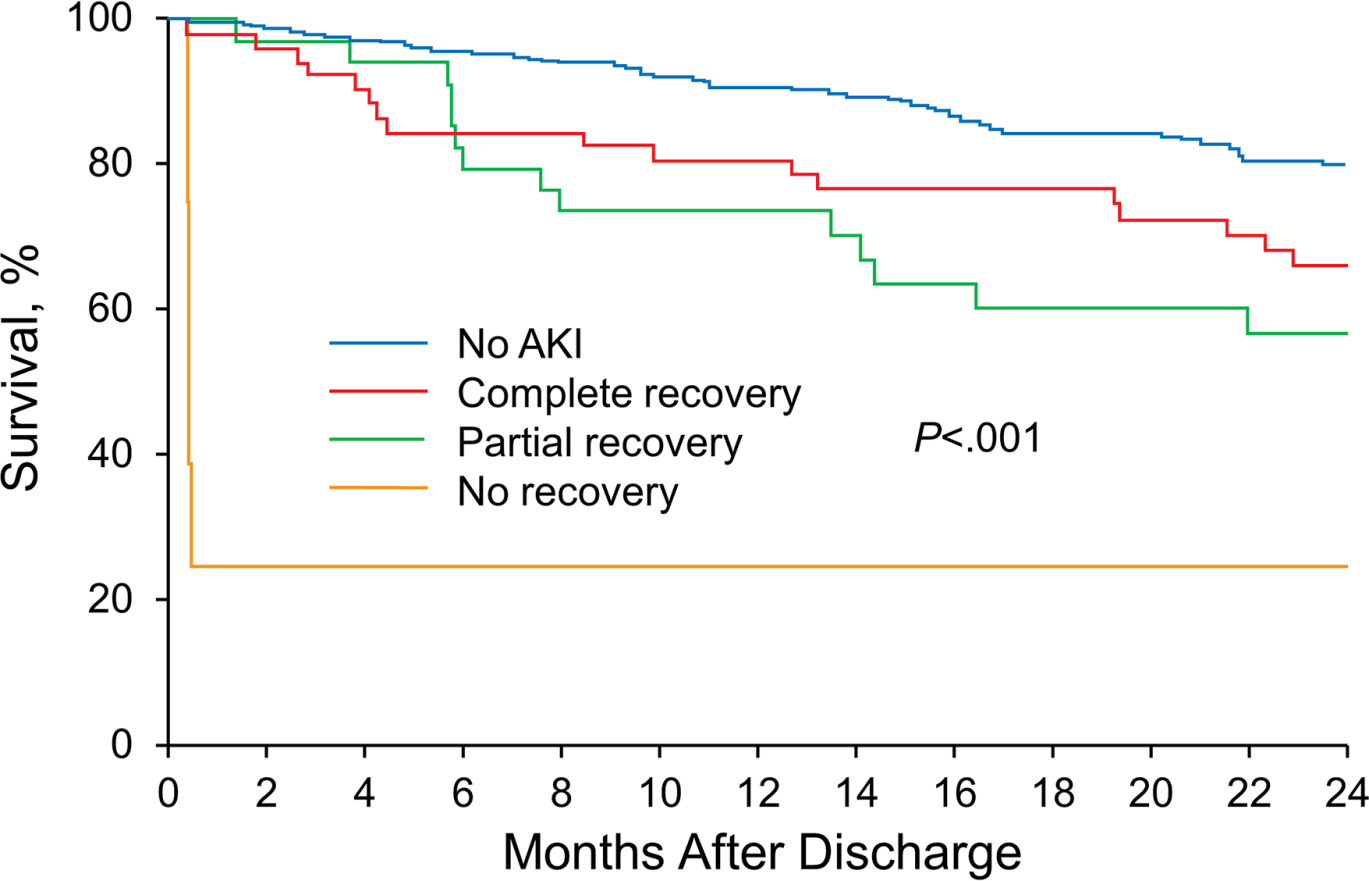
Kaplan-Meier curve showing 2-year follow-up stratified by recovery status after acute kidney injury (AKI).

During the study period, 98 (26%) patients developed AKI, of whom 55 (56%) had a complete renal recovery, 39 (40%) had partial renal recovery, and 4 (4%) had no renal recovery. The development of AKI was significantly associated with increased risk of 2-year mortality (hazard ratio [HR], 2.20 [95% CI, 1.37–3.49]). The 2-year mortality rate for patients with AKI was 34% for patients who had complete recovery, 43% for patients with a partial recovery, and 75% for patients with no renal recovery vs 20% for patients without AKI (*P* < .001) (Fig 1). In an adjusted analysis, complete recovery (HR, 1.87 [95% CI, 1.03–3.23]), partial recovery (HR, 2.65 [95% CI, 1.40–4.71]), and no recovery (HR, 10.95 [95% CI, 2.59–31.49]) after AKI were significantly associated with increased risk of 2-year mortality compared with complete recovery for patients who did not have AKI (Table 2). A subgroup analysis based on status of chronic kidney disease (GFR <60 mL/min/m^2^) and arterial approach showed similar results (Tables 3 and 4).

**Table 2:** Hazard ratios of renal recovery status for 2-year mortality.

Renal Recovery Status	2-Year Mortality, %	Hazard Ratio (95% CI)	*P* Value	Adjusted Hazard Ratio^a^ (95% CI)	*P* Value
No acute kidney injury	20	1 (ref)		1 (ref)	
Complete recovery	34	1.90 (1.06–3.22)	.03	1.87 (1.03–3.23)	.04
Partial recovery	43	2.59 (1.38–4.55)	.004	2.65 (1.40–4.71)	.004
No recovery	75	12.26 (2.98–33.45)	.002	10.95 (2.59–31.49)	.003

^a^ Adjusted for Society of Thoracic Surgeons risk score, age, estimated glomerular filtration rate, and arterial approach.

**Table 3:** Hazard ratios of renal recovery status for 2-year mortality: Subgroup analysis based on GFR.

Renal Recovery Status	2-Year Mortality, %	Hazard Ratio (95% CI)	*P* Value	Adjusted Hazard Ratio^a^ (95% CI)	*P* Value
**GFR <60 mL/min/m**^**2**^ **(n = 223**)					
No acute kidney injury	20	1 (ref)		1 (ref)	
Complete recovery	34	2.02 (1.01–3.83)	.047	1.84 (0.89–3.59)	.10
Partial recovery	48	2.97 (1.41–5.83)	.006	2.97 (1.36–6.11)	.01
No recovery	75	12.13 (2.89–34.58)	.003	9.81 (2.27–29.63)	.005
**GFR ≥60 mL/min/m**^**2**^ **(n = 151**)					
No acute kidney injury	21	1 (ref)		1 (ref)	
Complete recovery	31	1.68 (0.49–4.36)	.37	1.48 (0.33–4.56)	.56
Partial recovery	32	1.78 (0.42–5.11)	.38	1.86 (0.54–4.93)	.29
No recovery	…	…	…	…	…

Abbreviation: GFR, glomerular filtration rate.

^a^ Adjusted for Society of Thoracic Surgeons risk score, age, estimated glomerular filtration rate, and arterial approach.

**Table 4:** Hazard ratios of renal recovery status for 2-year mortality: Subgroup analysis based on arterial approach.

Renal Recovery Status	2-Year Mortality, %	Hazard Ratio (95% CI)	*P* Value	Adjusted Hazard Ratio^a^ (95% CI)	*P* Value
**Femoral Approach (n = 188)**					
No acute kidney injury	19	1 (ref)		1 (ref)	
Complete recovery	25	1.47 (0.43–3.76)	.49	1.22 (0.35–3.24)	.72
Partial recovery	33	2.24 (0.66–5.74)	.17	2.41 (0.71–6.21)	.14
No recovery	100	265.89 (24.62–5851.46)	< .001	227.87 (20.28–5130.88)	< .001
**Nonfemoral Approach (n = 186)**					
No acute kidney injury	22	1 (ref)		1 (ref)	
Complete recovery	39	1.97 (0.97–3.84)	0.06	2.20 (1.06–4.38)	.04
Partial recovery	48	2.64 (1.20–5.41)	0.02	3.13 (1.37–6.71)	.008
No recovery	50	3.96 (0.22–18.79)	0.27	4.33 (0.24–22.24)	.25

^a^ Adjusted for Society of Thoracic Surgeons risk score, age, estimated glomerular filtration rate

At 2 years after discharge, the eGFR of patients had changed as follows: no AKI, −2.7±13.9 mL/min/1.73 m^2^; complete recovery, −3.6±11.0 mL/min/1.73 m^2^; and partial recovery, −7.5±15.2 mL/min/1.73 m^2^. No significant differences were found in eGFR changes between groups (*P* = .18). No patients without AKI needed dialysis after discharge, whereas 2 patients who had complete or partial recovery required dialysis after discharge.

## Discussion

To our knowledge, this is the first report of rates of different renal recovery patterns after TAVR-associated AKI. Although most patients (56%) who developed AKI after TAVR completely recovered, 44% of patients did not have full recovery of their renal function (40%, partial recovery; 4%, no recovery) at hospital discharge. Long-term outcomes after TAVR were associated with development of AKI and subsequent recovery status at hospital discharge. Patients who did not develop AKI after TAVR had an 80% survival rate at 2 years. Of patients who developed AKI after TAVR, those who had a complete recovery had a 66% survival rate at 2 years, whereas the survival rate for those who did not recover from AKI was only 25%.

The Acute Disease Quality Initiative 16 Workgroup recently published a consensus report that emphasized the importance of renal recovery after AKI [14]. Recovery of renal function after AKI has been shown to be an independent determinant of morbidity and mortality in patients who are hospitalized, including in an intensive care unit, or who had cardiac surgery [17,26–28]. Studies done in intensive care units have shown that renal function did not completely recover in 8% to 26% of patients who had AKI by the time of hospital discharge [15,29–31]. In our study, which evaluated renal recovery in patients undergoing TAVR, we found that 44% of patients who had AKI following TAVR did not completely recover. This number is also higher than the previously reported nonrecovery rates among critically ill patients or patients undergoing cardiac surgery, which ranged from 9% to 39% (15, 28–31,32). However, patients who undergo TAVR have many comorbidities [7] that may affect the recovery of kidney function after AKI [31].

Previous studies showed that approximately 20% of survivors of AKI develop long-term complications characterized by chronic kidney disease, cardiovascular complications, physical limitations, disabilities, and greater mortality [32–35]. In our current study, survivors of AKI whose kidney function did not recover had an approximately 11-fold increased risk of 2-year mortality than those who did not have AKI. Our findings underscore the urgent need for better strategies to prevent AKI and to improve the care of patients with AKI who do not recover or only partially recover their kidney function. Because AKI after TAVR is multifactorial and related to pre-, intra-, and postoperative factors (such as patients’ comorbidities, baseline renal function, and catheter-based techniques), a multidisciplinary approach with careful risk stratification of patients and multiple targeted interventions should be incorporated into potential strategies to prevent TAVR-related AKI [36].

Our study has several limitations. The study design was retrospective and observational, which can create selection biases. In addition, the cohort was predominantly white, and a urine output criterion was not used for AKI diagnosis. Oliguria and increased creatinine levels are more common in patients with AKI who do not completely recover renal function [37]. Urinary output data were not used to identify AKI because the data were unavailable for most patients, and a substantial number of patients received diuretics after their procedure. A prospective, multicenter investigation is needed to address these limitations. Finally, the study had the disadvantage of our basing definitions of renal recovery on serum creatinine and/or eGFR because serum creatinine and true GFR have a nonlinear relation. Definitions of renal recovery based on serum creatinine might also be confounding [38].

## Conclusion

After TAVR, patients with AKI had an increased risk for mortality, and a reverse correlation existed between progressively higher risk of death and the extent of AKI recovery. Future studies are needed to identify better strategies to improve care for AKI survivors.

## Abbreviations

AKI: acute kidney injury
eGFR: estimated glomerular filtration rate
HR: hazard ratio
TAVR: transcatheter aortic valve replacement
VARC-2: Valve Academic Research Consortium-2
